# RNA Interference and Single Particle Tracking Analysis of Hepatitis C Virus Endocytosis

**DOI:** 10.1371/journal.ppat.1000702

**Published:** 2009-12-24

**Authors:** Kelly E. Coller, Kristi L. Berger, Nicholas S. Heaton, Jacob D. Cooper, Rosa Yoon, Glenn Randall

**Affiliations:** Department of Microbiology, The University of Chicago, Chicago, Illinois, United States of America; University of Washington, United States of America

## Abstract

Hepatitis C virus (HCV) enters hepatocytes following a complex set of receptor interactions, culminating in internalization via clathrin-mediated endocytosis. However, aside from receptors, little is known about the cellular molecular requirements for infectious HCV entry. Therefore, we analyzed a siRNA library that targets 140 cellular membrane trafficking genes to identify host genes required for infectious HCV production and HCV pseudoparticle entry. This approach identified 16 host cofactors of HCV entry that function primarily in clathrin-mediated endocytosis, including components of the clathrin endocytosis machinery, actin polymerization, receptor internalization and sorting, and endosomal acidification. We next developed single particle tracking analysis of highly infectious fluorescent HCV particles to examine the co-trafficking of HCV virions with cellular cofactors of endocytosis. We observe multiple, sequential interactions of HCV virions with the actin cytoskeleton, including retraction along filopodia, actin nucleation during internalization, and migration of internalized particles along actin stress fibers. HCV co-localizes with clathrin and the ubiquitin ligase c-Cbl prior to internalization. Entering HCV particles are associated with the receptor molecules CD81 and the tight junction protein, claudin-1; however, HCV-claudin-1 interactions were not restricted to Huh-7.5 cell-cell junctions. Surprisingly, HCV internalization generally occurred outside of Huh-7.5 cell-cell junctions, which may reflect the poorly polarized nature of current HCV cell culture models. Following internalization, HCV particles transport with GFP-Rab5a positive endosomes, which is consistent with trafficking to the early endosome. This study presents technical advances for imaging HCV entry, in addition to identifying new host cofactors of HCV infection, some of which may be antiviral targets.

## Author Summary

Hepatitis C virus (HCV) chronically infects 130 million people and is a major cause of cirrhosis and liver cancer. The current antiviral therapy of pegylated interferon-2 alfa + ribavirin is successful in only half of treated patients. This has led to an intensive effort to design improved therapeutic strategies. The identification of cellular cofactors of HCV infection greatly expands the pool of potential targets for drug design. In this paper, we combine RNA interference analysis of HCV endocytosis with the development of live cell imaging of highly infectious HCV particles. We identify 16 host cofactors of HCV entry, most of which function in sequential stages of clathrin-mediated endocytosis. We observe the trafficking of fluorescent HCV particles with these cellular cofactors and their related pathways, including the actin cytoskeleton, known receptors CD81 and the tight junction protein claudin-1, clathrin, an E3 ubiquitin ligase, and early endosomes. Surprisingly, given the role of tight junction proteins as HCV entry factors, virion entry generally occurred outside of cell-cell junctions. This paper identifies novel host targets for therapeutic development, describes techniques to image HCV entry, and provides insights into HCV-cell interactions in the entry process.

## Introduction

Hepatitis C virus (HCV) contains a small positive stranded RNA (∼9600 bp) genome encased by a capsid and surrounded by a lipid bilayer envelope containing the glycoproteins, E1 and E2 [1]. Delivery of the RNA genome to a protected site for replication depends on successful entry and trafficking in a hepatocyte. Studies using recombinant glycoproteins, HCV pseudoparticles (HCVpp), which are lentiviral vectors that incorporate the HCV glycoproteins E1 and E2 on the viral envelope, and more recently the infectious HCV cell culture system (HCVcc), have defined important HCV-receptor interactions [2]–[14]. Circulating HCV in the blood is associated with serum lipoproteins and requires an orchestrated engagement of receptors on target cells [15]–[19]. Initial attachment may be mediated by cell surface glycosaminoglycans and the LDL-receptor [8],[20],[21] of hepatocytes. Subsequent internalization of HCV requires a complex set of receptor molecules including, the tetraspannin molecule CD81, scavenger receptor B-1 (SR-BI), and the tight junction proteins claudin(s) (CLDN)-1, -6, -9, and occludin (OCDN) to bind and enter into hepatocytes [2]–[14], [22]–[26]. HCV internalization has never been visualized, but is postulated to occur at the tight junction where the entry receptors claudin-1 and occludin localize. Recombinant, soluble E2 re-localizes the CD81 receptor to the tight junction. Engagement of CD81 also activates the Rho GTPase, CDC42, which may result in actin cytoskeletal rearrangements that would allow for trafficking of HCV to the tight junction [27].

Small interfering RNAs (siRNAs) targeting the clathrin machinery inhibit HCV entry, suggesting that HCV uses clathrin-mediated endocytosis, however, HCV has not yet been visualized within a clathrin coated vesicle [28],[29]. Once internalized, it is thought that HCV traffics to the early endosome prior to viral RNA release [29]. Inhibitors of endosomal acidification block HCV entry, which indicates pH-dependent fusion between the envelope and endosome is needed to release the genomic content [2], [28]-[31]. Dominant–negative mutants of early, but not late, endosomal proteins block HCVpp entry, suggesting that HCV fuses from early endosomes [29]. These data lead to a model of HCV entry wherein the HCV virion initially engages the CD81 and SR-B1 co-receptors, then migrates to the tight junction where it interacts with its co-receptors, claudin-1, -6, or -9, and occludin. Clathrin-mediated endocytosis occurs at the tight junction and early endosomal acidification results in fusion, uncoating, and release of the HCV RNA genome into the cytosol [32]. Much of this model remains untested. In particular, we are interested in identifying the specific molecular requirements for HCV endocytosis and characterizing the trafficking of HCV virions in real time.

In this study, we have undertaken a combinatorial approach to understanding HCV entry into hepatocytes. We, and others, have previously relied on RNA interference (RNAi) analysis to identify cellular cofactors of HCV replication [33]–[38]. These studies have generally used HCV sub-genomic replicons, which do not produce infectious virus and as such, cannot be used to study HCV entry or egress [7]. The only siRNA study using infectious HCV focused on cellular proteins that interact with HCV and identified few targets of interest for HCV endocytosis [35]. It is likely that the major virus-cell interactions that were missed in these analyses are the roles of host membrane trafficking pathways in HCV infection, since this encompasses various endocytic and secretory pathways. Therefore, we began this study by evaluating a siRNA library targeting 140 genes involved in membrane trafficking pathways to identify human genes required for infectious HCV production and HCVpp entry. From the siRNA study, we identified several cellular genes involved in the clathrin-mediated endocytosis pathway. Next, we established single particle tracking approaches to image fluorescent HCV particles in live cells, based on a similar approach used with Dengue virus [39]. We demonstrate co-localization and co-trafficking of HCV virions with numerous cellular markers identified in the RNAi analysis. Here, we provide evidence that HCV traffics initially with filopodia, then its co-receptors, CD81 and claudin-1, and is internalized into Huh-7.5 cells at regions not restricted to the tight junction. Internalization is associated with the clathrin light chain, actin, and the E3 ubiquitin ligase c-Cbl. Intracellular HCV is associated with early endosomes positive for Rab5a GTPase and are likely to fuse out of endosomes, dependent on a vacuolar ATPase, encoded by the *ATP6V0A1* gene.

## Results

### Host genes involved in HCV endocytosis

We began this study by interrogating a previously described unique siRNA library that targets known membrane trafficking pathways [33]. The base of this library is a commercially available set of 122 siRNAs targeting membrane trafficking genes from Dharmacon, Inc. that was supplemented with genes involved in fatty acid synthesis, COPI vesicles, ADP ribosylation factors, autophagy, and secretion. Genes targeted in this library also include those involved in clathrin-mediated endocytosis, caveolae, macropinocytosis, cytoskeletal organization, and phosphatidyl inositol signaling. Analysis of this library provides an unbiased assessment of the relative contributions of these pathways to HCV endocytosis.

We first identified siRNAs that inhibited infectious HCV production and then re-tested these siRNAs using HCVpp that contain a luciferase reporter gene. The rationale for this approach was to do a primary evaluation of host cofactors using the most authentic system (HCVcc) and then distinguish host genes involved in HCV endocytosis from those acting at later stages of the viral life cycle by using HCVpp. Huh-7.5 cells were transfected with a pool of four siRNAs that target a specific host gene, incubated 72 hours to establish silencing, then inoculated for 16 h with HCV. Infection was maintained for a total of two days, after which supernatants were collected and infectious HCV was quantified in duplicate by limiting dilution titration. Infectious HCV production following siRNA treatment was then normalized to the amount of HCV produced following treatment with four independent irrelevant siRNAs (Table 1). siRNAs targeting 44 genes altered infectious HCV production greater than one standard deviation from the irrelevant siRNAs (greater than 2.8-fold) and these were prioritized for follow up in the HCVpp entry assay. The siRNA pools were individually introduced into Huh-7.5 cells and subsequently infected with HCVpp. Luciferase values were measured 48 hours after HCVpp infection and normalized to values of cells transfected with irrelevant siRNAs. siRNAs that inhibited HCVpp luciferase expression by greater than one standard deviation (1.35-fold) were classified with a defect in HCV endocytosis. Specificity controls for the RNAi study included measuring cell viability (Figure S1), demonstrating that multiple siRNAs produce a consistent phenotype (Table S1), and confirming that the target genes were indeed silenced by the indicated siRNA using gene-specific real time RT-PCR assays (Table S2). In total, 16 host genes were identified that satisfied all criteria (Table 1).

**Table 1:** Cellular cofactors of HCV endocytosis.

siRNA	Percent Inhibition†			Gene Function
	HCVpp	HCVcc	Host Gene RNA	
CLTCL1	73+6.4	69+12	69+1.3	clathrin coat
CLTB	63+4.1	84+12	91+1.2	clathrin coat
HIP1R	26+3.6	66+17	74+1.9	clathrin coat
HIP1	48+6.2	81+15	52+4.4	clathrin coat
EPN1	35+4.7	71+12	77+0.2	clathrin/actin
EPN3	28+4.3	84+8.6	69+2.8	clathrin/actin
CFL1	85+4.4	89+8.1	86+1.7	actin polymerization
CDC42	56+6.8	78+16*	95+0.3	actin polymerization
ROCK2	41+3.7	77+19*	77+0.2*	actin polymerization
AP2M1	39+5.2	−79+15‡	88+2.0	AP2 adaptor
SYT1	64+7.9	72+13	65+0.4	AP2/clathin recruitment
CBL	57+7.3	72+12	WB	ubiquitin ligase
HGS	28+5.2	71+12	70+0.6	ubiquitylated receptor sorting
ATP6V0A1	54+6.5	76+13	59+3.8	endosomal acidification
STAU	50+7.1	72+13	N/A	ER/Golgi trafficking
RAB7L1	53+6.4	81+8.3*	85+5.4*	Rab GTPase

**†:**  = Values represent % inhibition plus SEM of the geometric mean as compared to irrelevant siRNA treatment.

**‡:**  = A negative value reflects an increase in secretion of infectious virus.

*:  = Values previously published in reference [33].

WB = confirmed by western blot; N/A = not available.

Overall, the RNAi data implicate the clathrin-mediated endocytosis pathway, including genes that encode clathrin coat components (CLTB, CLTCL1, HIP1, and HIP1R); AP-2 adaptor (AP2M1); AP-2-clathrin recruitment during endocytosis (SYT1) [40]; proteins linking clathrin and the actin cytoskeleton (EPN1 and EPN3); and proteins controlling actin polymerization (CFL1, CDC42 and ROCK2). c-CBL encodes an E3 ubiquitin ligase linked to receptor endocytosis and receptor tyrosine kinase signaling. Hgs is involved in the sorting of ubiquitylated receptors and invagination of endosomal membranes during trafficking from the early endosome to the late endosome or multivesicular body [41]. The vacuolar ATPase, ATP6v0a1, is involved in endosomal acidification and may function in the pH-dependent uncoating of the HCV genome. The requirement of host proteins that function in clathrin-dependent endocytic pathways and endosomal acidification for HCV endocytosis is supported by previous studies demonstrating that inhibition of these processes by chlorpromazine (clathrin coated pit inhibitor), bafilomycin A1 (vesicular protein ATPase inhibitor), ammonium chloride or chloroquine (inhibit endosomal acidification) inhibit HCV entry [28],[29],[31]. siRNAs targeting CDC42, ROCK2, and Rab7L1 inhibit sub-genomic HCV replication in addition to HCV entry [33], suggesting multiple roles for these genes in the HCV life cycle.

### Production of highly infectious fluorescent HCV particles

**Figure 1:**
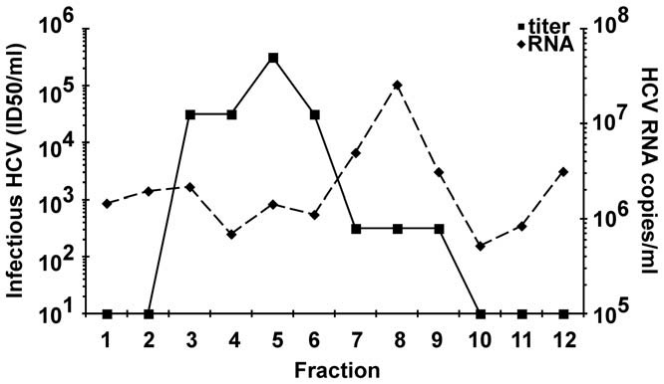
Purification of highly infectious DiD-HCV particles. Polyethylene glycol concentrated HCV was labeled with DiD (5 µM), layered on a 20–80% sucrose gradient, and purified by ultracentrifugation. Top of the gradient is fraction 1. Infectious HCV (solid line, left y-axis) was quantified by titration, while HCV RNA copy numbers (dashed line, right y-axis) were quantified by real time RT-PCR. Note the different scales for each y-axis.

We next developed approaches to visualize the association of HCV virions with the host endocytic machinery. To detect single HCV particle entry events in Huh-7.5 cells, we labeled virions with the membrane-permeable lipophilic dyes, DiD or DiI. Previous reports show this approach was successful in labeling Dengue virus and influenza virus for single particle imaging in live cells without affecting viral infectivity [39], [42]-[44]. Incorporation of DiD did not have deleterious effects on HCV infectivity (Table 2). The specific infectivity of HCV that is isolated from cell culture supernatants is approximately 1000 (where 1 in 1000 HCV particles lead to a productive infection) [7]. Poor specific infectivity limits the ability to track fluorescent HCV particles with confidence that they represent a productive infection. Therefore, we purified a highly infectious fraction of DiD-labeled HCV (DiD-HCV). We exploited the property that HCV isolated from cell culture supernatants and patients possesses a range of densities [45]. The different densities are attributed to the association of low-density and very-low-density lipoprotein with virions [7],[15]. The majority of HCV RNAs sediment at a density of ∼1.15 g/ml, while a highly infectious fraction of HCV is associated with a lower density (∼1.06 g/ml) [7],[19],[45]. DiD-HCV was separated by sucrose gradient density ultracentrifugation, fractions were collected, and the fraction density, infectious HCV and HCV RNA copy numbers were quantified (Figure 1). Purified DiD-HCV had a significantly improved specific infectivity of 10.5±8.5 (Table 2). This improved specific infectivity greatly increases confidence that we can visualize relevant HCV entry events; however, there remains a caveat that ∼90% of the purified, labeled particles do not lead to productive infection.

**Table 2:** Fluorescent HCV infectivity.

Virus	Specific Infectivity (SI)
HCV	2009 (±450)
DiD-HCV	1018 (±346)*
Purified DiD-HCV	10.5 (±8.5)**

*: no significant difference between HCV and DiD-HCV (*p* = .52).

**: average density = 1.06±.03 g/mL.

**Figure 2:**
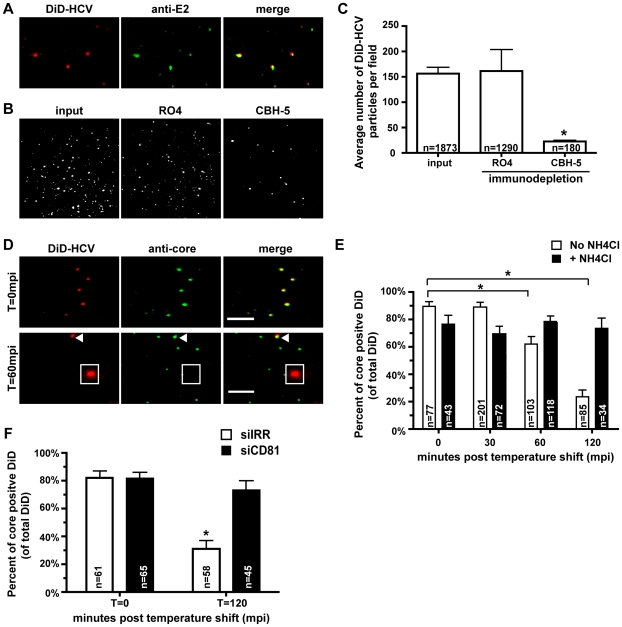
Characterization of DiD-HCV particles. A. DiD-HCV particles from the high specific infectivity fraction (density ∼1.06 g/mL) were spotted onto poly-lysine treated coverslips and immunostained with the HCV E2 monoclonal antibody, CBH5. B. DiD-HCV was immunodepleted using anti-E2 (CBH5) or isotype control (RO4). 10 µL of unbound particles were spotted onto coverslips and fluorescence images were acquired. C. DiD signal from (B) was quantified using the Image J plugin, measure particles. D. Huh-7.5 cells were infected with DiD-HCV for 1 hour on ice and fixed in 4% paraformaldehyde. The DiD-HCV was probed with HCV core monoclonal antibody. Shown are images of DiD-HCV envelope and core (capsid) colocalization at T = 0 (top) and T = 60 mpi post temperature shift (bottom). Arrow denotes DiD-core colocalization at 60 mpi, whereas the square denotes DiD no longer co-localizing with HCV core. E. Co-localization of DiD-envelope and core as determined by immunofluorescence was performed over time in the presence or absence of the endosomal inhibitor NH_4_Cl (100 mM). Co-localization was quantified at the indicated time points. Values are reported as percent of DiD particles positive for core staining. n = total DiD signal. Error bars represent standard error of proportions for percent co-localization. Asterisks indicate means are significantly different from T = 0 time point for untreated cells, *p* = 0.0001. F. Huh-7.5 cells were electroporated with irrelevant (siIRR) or CD81 (siCD81) siRNAs, maintained for two days and then the uncoating assay was performed as in (E), *p* = 0.0001.

The majority of DiD-labeled viral particles have an equivalent size based on fluorescence emissions, suggesting we were imaging single viral particles (Figure 2A). Additionally, the number of fluorescent DiD particles per cell corresponded to our predicted number of HCV virions, based on HCV RNA copy number quantitation of the inoculum. To confirm the DiD signal is specific to labeled HCV particles, we immuno-stained DiD-HCV particles from our highly infectious fraction with an E2 antibody (CBH-5) which recognizes E2 on extracellular released virions [46],[47]. Co-localization between DiD and E2 was quantified (Figure 2A). Overall, 95.7±1.3% of DiD particles immuno-stained for E2. Next we tested if the DiD-HCV particles could be depleted in the presence of the anti-E2 antibody, CBH-5. Anti-E2 immuno-depletion resulted in an 86.6% reduction in DiD signal compared to input inoculum, while the isotype control antibody (R04) did not alter DiD signal (Figure 2B and 2C).

We next quantified DiD-core co-localization over a time course of infection to determine the kinetics of virion uncoating. In this assay, uncoating is defined as DiD-labeled envelope that no longer co-localizes with the HCV capsid. Huh-7.5 cells were infected at 4°C for 1 hour to allow binding of the virus, shifted to 37°C to allow HCV entry, fixed and processed for immunofluorescence detection of core protein (Figure 2D). 89.6±3.48% (n = 77) of DiD labeled particles were core positive at time zero, indicating efficient labeling of the virus. We found that the majority of uncoating is complete within two hours of the temperature shift, with 50% of uncoating occurring 85 minutes after temperature shift (Figure 2E). The kinetics of DiD-HCV uncoating are similar to what has been reported with HCVpp [29]. To test if the DiD-HCV particles enter through pH-dependent endocytosis, we performed the uncoating assay in parallel in the presence of NH_4_Cl, which neutralizes acidic endosomal compartments and has previously shown to inhibit HCV entry [31]. DiD-core colocalization did not significantly decrease at times post temperature shift in NH_4_Cl treated cells, indicating that DiD-HCV uncoating requires endosomal acidification (Figure 2E).

Finally, we tested whether DiD-HCV entry and uncoating requires the receptor, CD81 [9]. We treated Huh-7.5 cells with either irrelevant or CD81 siRNAs for two days and then performed the uncoating assay (Figure 2F). We observed significant DiD-HCV uncoating in the irrelevant (siIRR) treated cells (from 82% DiD-core co-localization at T = 0 to 31% DiD-core co-localization at T = 120 minutes). In contrast, CD81 siRNAs inhibited DiD-HCV uncoating. We found only a modest reduction in DiD-HCV uncoating in the CD81 silenced cells (from 81% DiD-core co-localization at T = 0 to 73% DiD-core co-localization at T = 120 minutes). Thus, DiD-HCV entry and uncoating requires CD81. Taken together, these experiments demonstrate that DiD-HCV particles efficiently co-localize with viral structural antigens and have the same cellular requirements for entry, CD81 and endosomal acidification, as does native HCV.

### Localization of HCV with the actin cytoskeleton

**Figure 3:**
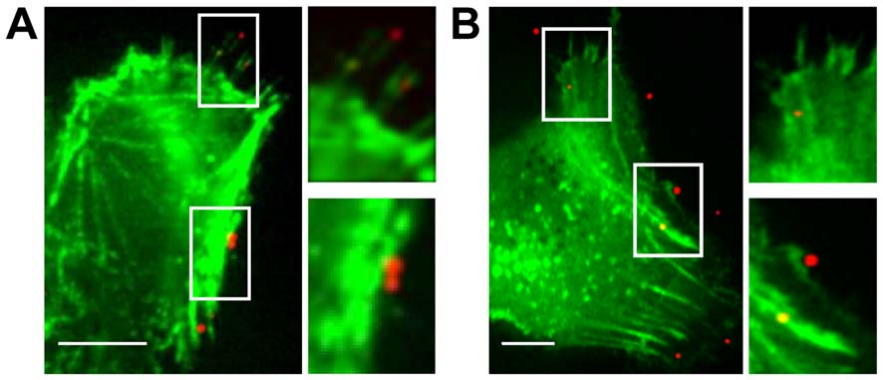
HCV localizes to actin rich filopodia and stress fibers during entry. DiD-HCV was allowed to bind to Huh-7.5 cells on ice for 1 hour followed by temperature shift to 37°C. Cells were fixed at 30 minutes post temperature shift (A) or 60 minutes post temperature shift (B) and stained for actin using Alexa Fluor 488 phalloidin (A) or were expressing GFP-actin (B). Enlarged insets are shown on the right. Scale bars = 10 µm. A. Inset shows DiD-HCV association with filopodia (top) and cortical actin cytoskeleton. B. Inset shows DiD-HCV association with actin stress fibers along the plasma membrane.

Our siRNA studies indicated a role for actin and clathrin-actin association for efficient HCV endocytosis. Several viruses have been shown to rely on retrograde transport from actin-rich filopodia or retraction fibers to the cell body before becoming internalized [48]-[51]. The role of actin in HCV entry was investigated by fluorescence microscopy in both fixed and live cells. Huh-7.5 cells were infected with DiD-HCV on ice for 1 hour, then shifted to 37°C, fixed at various time points after the temperature shift, and stained for F-actin (Figure 3A). At early time points, fluorescent viral particles were found associated with filopodia protruding from the cell surface. Viral particles were found associated with the base of the filopodia during the first 30 minutes post temperature shift and decreased at later time points. DiD-HCV particles also associated with cortical actin and actin stress fibers at later time points (Figure 3B). We did not observe any significant morphological changes to the cortical actin cytoskeleton compared to uninfected cells during entry time points as previously reported [27].

### Live cell imaging of HCV and actin surface dynamics

**Figure 4:**
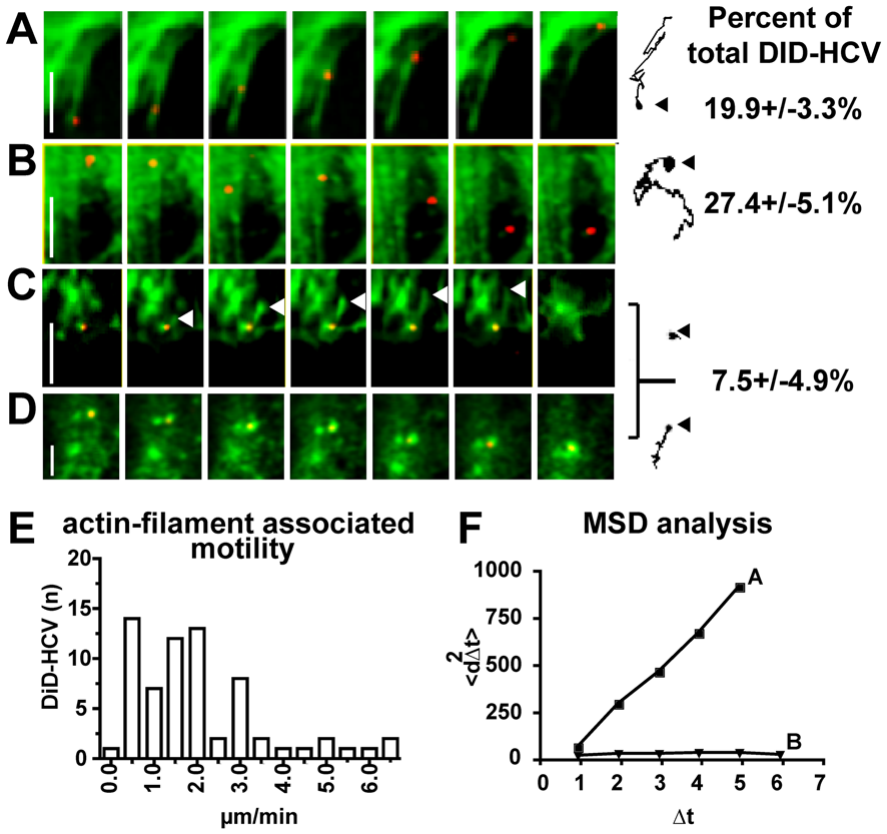
HCV entry dynamics in association with the actin cytoskeleton. Huh-7.5 cells transiently expressing GFP-actin (green) were infected with DiD-HCV on ice for 1 hour then shifted to 37°C upon imaging. Shown are representative montages of alternating time-lapse exposures of Cy5 (DiD-HCV) and GFP (actin) during entry. Scale bar = 10 µm. A. DiD-HCV uses filopodia-associated transport to reach the cell body. *Right*: Trajectory tracing depicting the path followed by the DiD-HCV particle during imaging. Arrow points to the origin of transport for the trajectory. Scale bar = 10 µm. B. DiD-HCV particle displaying random movement not associated with actin. *Right*: Trajectory of particle shown in montage. Scale bar = 10 µm. C. DiD-HCV entry is associated with actin nucleation. White arrows point to tip of nucleating actin filament. Particle trajectory is shown to the right. Scale bar = 10 µm. D. Actin nucleation event leading to active transport of DiD-HCV. Scale bar = 5 µm. Numbers on the far right indicate the % of DiD-HCV particles (of total DiD-HCV bound to cells) displaying each movement. The remaining 43.8±7.8% of DiD-HCV particles displayed no movement at time of recording. E. DiD-HCV particles associated with actin stress fibers during the duration of imaging were tracked and the velocities (µm/min) were determined. The velocities were binned from 67 DiD-HCV particles and plotted by the number of DiD-HCV particles exhibiting a certain speed. F. Mean square displacement (MSD) plots of the trajectories from A and B showing active transport (A) and diffusion (B).

We next examined the dynamic associations of HCV with actin during endocytosis in live cells. We infected Huh-7.5 cells that were transiently expressing GFP-actin [52] at multiplicities of infection that result in approximately four bound DiD-HCV particles per cell. Infections were maintained on ice for 1 hour to allow for binding but not internalization, and then unbound particles were washed off. DiD-HCV transport was visualized every 5 seconds for several minutes following a shift to 37°C. Individually moving DiD-HCV particles were tracked and the trajectories of particle movements were determined (Figure 4).

Bound DiD-HCV particles exhibited heterogeneous movement where many particles had limited mobility, while some displayed highly active trajectories. Distinct particle trajectories included processive linear movement, random movement, or restricted movement in a minimal area (Figure 4A, B, or C, respectively). 85%±3.6% (n = 55) of motile particles were associated with actin during transport. At early time points post temperature shift, DiD-HCV particles localized with actin-rich filopodia. Figure 4A (Video S1) depicts a DiD-HCV particle bound to a filopodia, followed by transport of the particle towards the plasma membrane at an average velocity of 0.082±0.008 µm/sec, which is consistent with retrograde actin flow. Overall, DiD-HCV particles that associate with actin filaments move at an average velocity of 2.07±1.5 µm/min (Figure 4E). DiD-HCV particles that associated with the plasma membrane, but not filopodia or actin stress fibers, either moved randomly or were relatively static (Figure 4B, Video S2). This result was surprising, since engagement of CD81 with antibody or soluble HCV E2 has been reported to initiate a directed migration to cell-cell junctions that is associated with actin [27]. Therefore, we performed a quantitative mean square displacement (MSD) analysis of HCV movements at the cell surface. DiD-HCV particles moving in association with filopodia or stress fibers possess a MSD curve associated with active transport (Figure 4F, sample A). In contrast, DiD-HCV particles at the plasma membrane, which are only transiently localized with actin, have a MSD curve representative of diffusion (Figure 4F, sample B). It is possible that DiD-HCV displays diffuse movement on the cell surface until a clathrin-coated pit is found for internalization to occur, as has recently been shown for the related Dengue virus [42].

Static, surface-bound HCV particles were either immobile (and possibly non-infectious); or alternatively, they were associated with actin rich clusters that occasionally nucleated from the DiD-HCV particle. An example of actin nucleation associated with particle internalization is shown in Figure 4C (Video S3), wherein an actin filament nucleates adjacent to the DiD-HCV particle, then surrounds the base of the particle, followed by the particle leaving the focal plane, likely due to internalization. This is reminiscent of the clustering of actin around a clathrin-coated pit during endocytic internalization [53],[54]. Additionally, DiD-HCV particles were found to transport with nucleating actin clusters (Figure 4D, Video S4).

### HCV is associated with actin following internalization

**Figure 5:**
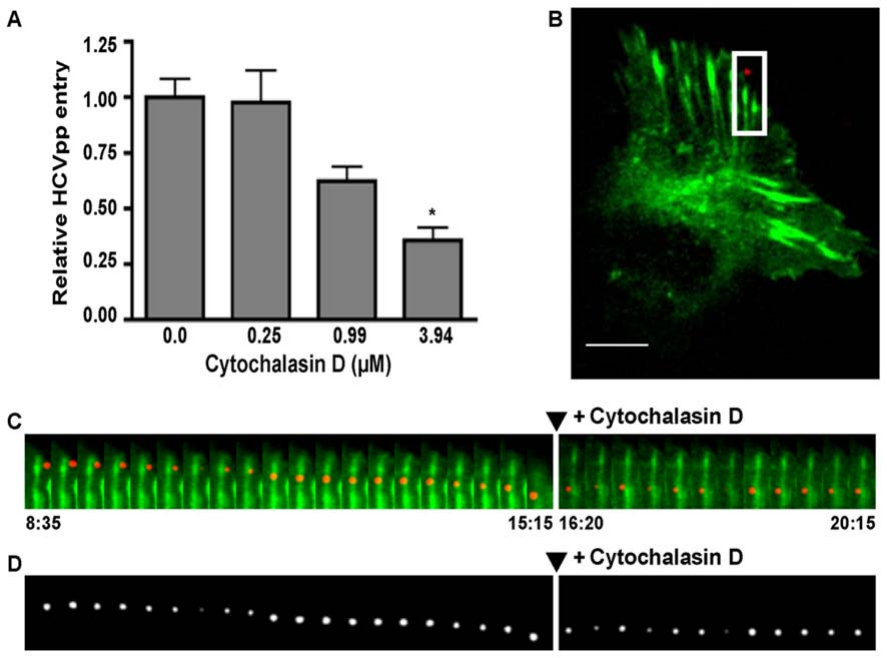
HCV entry requires an intact actin cytoskeleton. A. Huh-7.5 cells were incubated with indicated concentrations of cytochalasin D for one hour and infected with HCV pseudoparticles (HCVpp) expressing the luciferase reporter gene for four hours. Luciferase measurements were taken at 48 hours post infection. Plotted are relative luciferase values normalized to DMSO vehicle control. Cell viability was unaffected (data not shown). Asterisk indicates *p*<0.05, indicating significance. B. Huh-7.5 cells transiently expressing GFP-actin (green) were infected with DiD-HCV on ice for 1 hour then shifted to 37°C upon imaging. Scale bar = 10 µm. C. Montage of alternating time-lapse exposures of Cy5 (DiD-HCV) and GFP (actin) from inset in (B). Height of montage = 11.36 µm. Cells were treated with 2 µM cytochalasin D to depolymerize actin filaments at time indicated (arrow). Indicated times are in minutes: seconds. D. Time-lapse montage of (C), Cy5 (DiD-HCV) only. Experiment is representative of multiple replicates.

We next examined HCV-actin interactions following internalization (Figure 5B–D). In the first 10 minutes of imaging, the plasma membrane ruffles around the particle followed by internalization into the cytoplasm. Once inside the cell, the viral particle migrates toward a pre-formed actin stress fiber and is then transported along the stress fiber at an average velocity of 1.8 µm/min. To confirm that this was indeed actin-based motility, we tested whether treatment with cytochalasin D would interfere with the entry process. We first showed that pretreatment of Huh-7.5 cells with cytochalasin D produces a dose-dependent inhibition of HCVpp entry (Figure 5A). Then, we treated the infected cells shown in Figure 5B with 2 µM cytochalasin D at 20∶15 (minutes: seconds) of imaging (Figure 5C, Video S5). Treatment stalled DiD-HCV migration along the stress fiber for the remainder of imaging, as shown in Figure 5C. To determine whether particles associated with actin stress fibers were internalized, we treated DiD-HCV infected cells with 0.2% trypan blue to quench the DiD fluorescence of extra-cellular DiD-HCV particles. We observe that stress fiber-associated viral particles were resistant to fluorescence quenching, indicating they are internalized. 75% (SEp±6.5, n = 44) of fluorescent DiD-HCV particles after quenching were associated with phalloidin stained actin stress fibers. Also we did not detect DiD-HCV particles associated with filopodia after quenching with trypan blue, indicating that these DiD-HCV particles are extra-cellular (data not shown).

### HCV is associated with the clathrin-mediated endocytosis machinery

**Figure 6:**
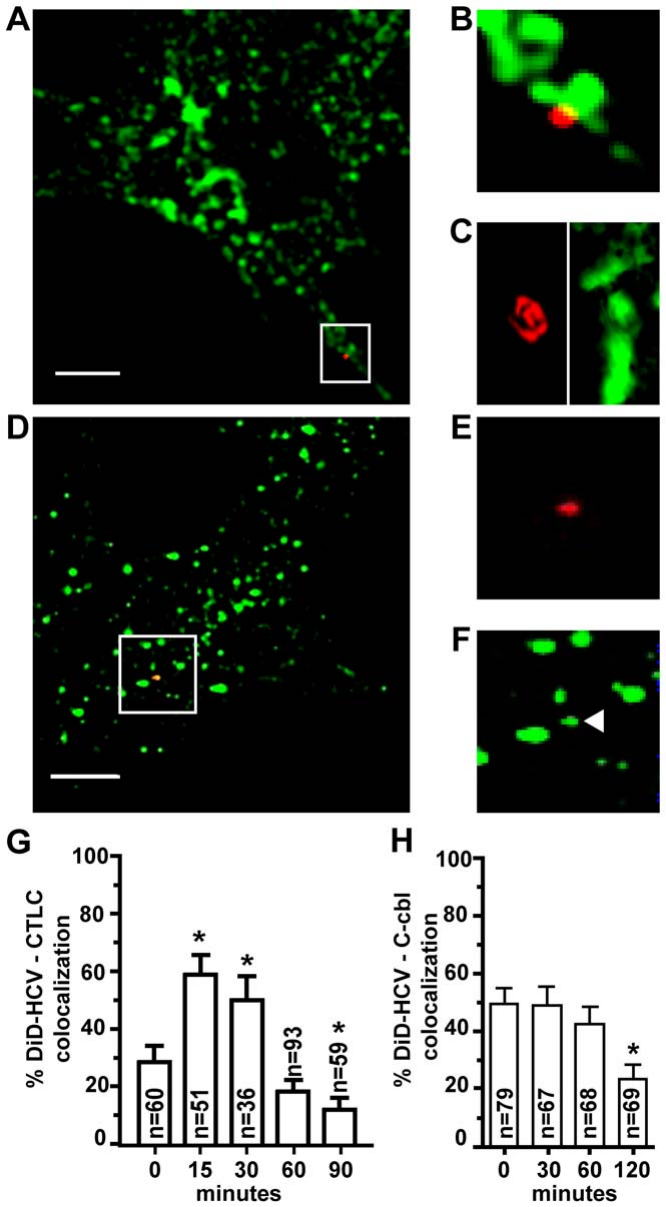
HCV entry is associated with the clathrin endocytosis machinery in hepatocytes. Huh-7.5 cells were infected with DiD-HCV for 1 hour on ice then shifted to 37°C. Cells were fixed and stained for immunofluorescence using clathrin light chain and c-Cbl antibodies. A. Merge image of DiD-HCV (red) on cell surface with clathrin light chain (green). B. Enlarged image inset of (A). C. 3-dimensional Z-stack reconstruction of (A). D. Merged image of labeled DiD-HCV (red) with c-Cbl (green). E. Enlarged image inset of (D) DiD-HCV only. F. Enlarged image inset of (D) c-Cbl only, arrow denotes colocalized c-Cbl. Scale bar = 10 µm. G. Percent of DiD-HCV particles colocalized with clathrin light chain (CTLC) at the indicated minutes post temperature shift. H. Percent of DiD-HCV particles colocalized with c-Cbl at the indicated minutes post temperature shift. n = total DiD signal. Asterisk indicates *p*<0.05.

Our RNAi data implicate clathrin-mediated endocytosis in HCV entry, as has been previously reported using siRNA and inhibitor studies. [28],[29],[31]. We tested the association of HCV with clathrin by infecting Huh-7.5 cells with DiD-labeled virus at 4°C for 1 hour, then shifted to 37°C and fixed at various time-points after the temperature shift. Cells were immuno-probed for clathrin light chain, a component of clathrin triskelions (Figure 6A). Z-stack analysis shows clathrin light chain associated beneath a DiD-HCV particle (Figure 6C). 28.3% (SEp±5.8%, n = 60) of cell-associated DiD-labeled HCV particles co-localized with clathrin light chain at zero minutes post infection whereas maximal association 58.8% (SEp±6.9%, n = 58) of cell associated particles occurred around 15 minutes post infection (Figure 6H). In a recent report from Schwarz et al, HCV bound to cells is internalized within 23 minutes and is consistent with the time points when we observe maximal association with clathrin light chain (Figure 6H) [55].

The E3 ubiquitin ligase, c-Cbl, was identified as an entry cofactor in our siRNA screen and has been shown to mediate receptor internalization by ubiquitylating the cytosolic domains of multiple receptor molecules [56]. We examined whether there was a direct association between DiD-HCV and c-Cbl during entry to eliminate the possibility of an indirect phenotype. Cells were infected with DiD-HCV as above, fixed at various times points, immuno-probed for c-Cbl, and visualized by confocal microscopy. We observed specific co-localization of DiD-HCV with c-Cbl (Figure 6D). Maximal association of DiD with c-Cbl occurred at time points prior to HCV uncoating (Figure 6I & 2F).

### HCV entry in Huh-7.5 cells is not preferentially associated with cell-cell junctions

**Figure 7:**
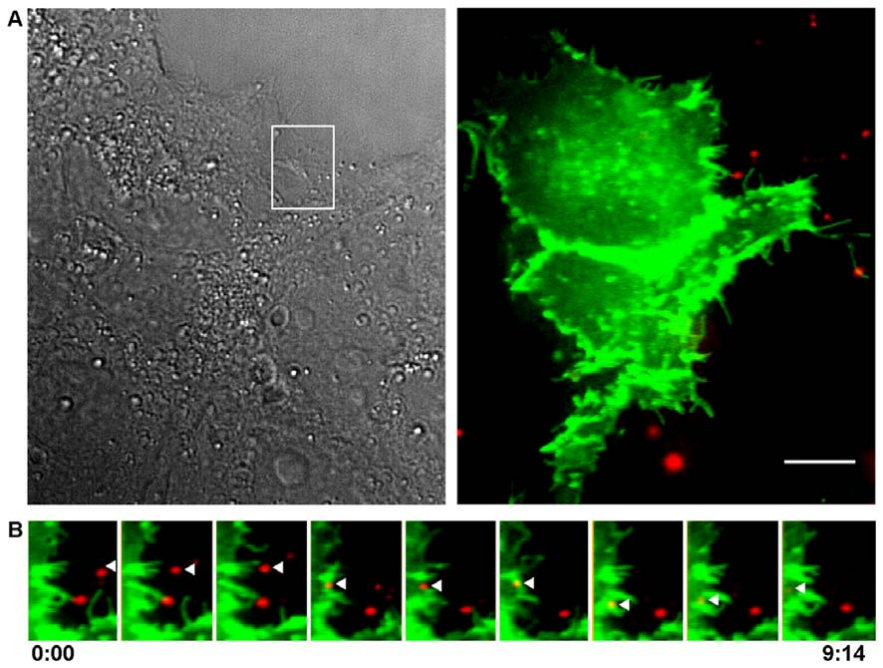
HCV enters cells in association with CD81. Huh-7.5 cells expressing CD81-GFP were infected with DiI fluorescently labeled HCV (red) on ice. Cells were shifted to 37°C on the microscope stage. A. *Left*: DIC image. *Right*: Merge image of GFP and DiI fluorescence. The box highlights the live cell montage shown in panel (B). B. Montage of alternating time-lapse exposures of Cy3 (DiI-HCV) and GFP (CD81-GFP). Alternating 100ms exposures of Cy3 and GFP were taken every 10 seconds for several minutes post temperature shift. Time is in minutes: seconds. Scale bar = 10 µm.

We next visualized the association of DiI-HCV with one of the known entry receptors, the tetraspannin CD81. Huh-7.5 cells were infected with CD81-GFP lentivirus and maintained for two days to establish CD81-GFP expression. CD81-GFP had characteristic localization to the plasma membrane. DiI-labeled HCV was allowed to bind to the surface of Huh-7.5 cells on ice then shifted to 37°C upon imaging. As shown in Figure 7 (Video S6), a viral particle migrated toward the cell surface, engaged CD81-GFP on the cell surface, and was internalized within nine minutes. Notably, this internalization occurs outside of the area of cell-cell contact, as indicated by the DIC image and the area of intense CD81-GFP fluorescence (Figure 7A).

**Figure 8:**
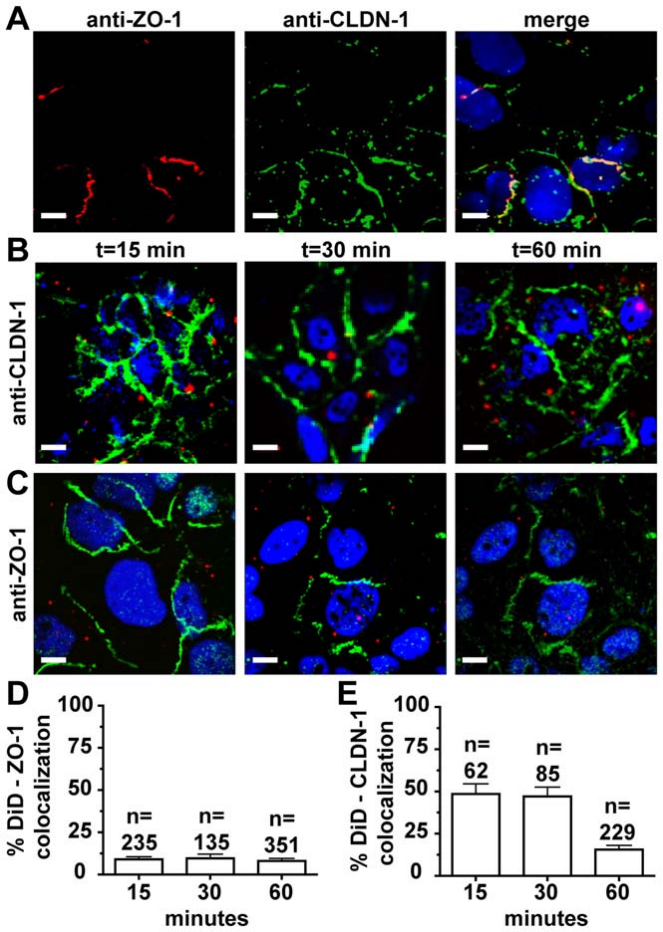
HCV-Claudin-1 association outside of cell-cell junctions. Huh-7.5 cells were stained by immunofluorescence for the tight junction proteins, zona occludens-1 (ZO-1), and claudin-1 (CLDN-1) in the presence or absence of DiD-HCV infection. A. CLDN-1 staining is not restricted to areas of ZO-1 localization. Huh-7.5 cells grown to confluency were fixed in 4% paraformaldehyde and stained for CLDN-1 and ZO-1. B. Huh-7.5 cells were infected with density gradient purified DiD-HCV for 1 hour on ice. Cells were shifted to 37°C, fixed in 4% paraformaldehyde at various time points post temperature shift, and stained for CLDN-1 by immunofluorescence. Shown are cells fixed at 15, 30, and 60 minutes post temperature shift. Nuclei are stained with DAPI (blue). C. Localization of DID-HCV (red) with the tight junction marker, ZO-1 (green). Shown are cells fixed at 15, 30, and 60 minutes post temperature shift. D. Quantification of percent co-localized DiD-HCV positive for ZO-1 fluorescence at the indicated minutes post temperature shift. E. Quantification of percent colocalized DiD-HCV positive for CLDN-1 fluorescence at the indicated minutes post temperature shift. n = total DiD signal. Scale bar = 10 µm.

The current model of HCV entry supports trafficking of the virus after engagement of the CD81 receptor on the cell surface to the tight junction, where engagement of the co-receptors claudin-1 and occludin permits internalization. Our live cell imaging approaches consistently failed to show trafficking of DiD-HCV to sites of cell-cell contacts. Instead, virus internalization occurred at various sites on the cell surface. We decided to look at the localizations of endogenous receptor proteins in fixed cells. Immunofluorescence of Huh-7.5 cells for claudin-1 and the tight junction marker zona occludins (ZO-1) show that claudin-1 co-localizes with ZO-1, in addition to localizing to various cell surface locations (Figure 8A). We then investigated the apparent lack of DiD-HCV re-localization to cell-cell contacts in fixed cells over a time course so that we could increase our quantitation of DiD-HCV localization. DiD-HCV infection was synchronized on ice, unbound particles were removed, and cells were fixed at various time points following temperature shift to determine if viral particles accumulate at site of cell-cell contacts (Figure 8B–C). The number of particles localized to cell-cell junctions was low (∼8%) and did not increase with time (Figure 8D). In contrast, the co-localization of DiD-HCV with claudin-1 was much greater at 15 and 30 minutes after temperature shift (∼48%) (Figure 8B and E). Thus, the majority of DiD-HCV-claudin1 interactions occur outside of cell-cell junctions in Huh-7.5 cells. DiD-HCV-claudin1 co-localization dropped dramatically at 60 minutes after temperature shift, suggesting that the majority of particle internalization occurred before this time point (Figure 8E). The lack of tight junction re-localization may reflect the fact that Huh-7.5 cells are poorly polarized, with the viral receptor claudin-1 available on the plasma membrane outside the tight junction. Thus, in primary polarized hepatocytes, entry may occur at the tight junction, but our data indicates HCV entry *in vitro* does not require internalization at a tight junction.

### DiD-HCV particles traffic to Rab5a-positive endosomes

We next determined if the DiD-HCV particles displaying motility on the cell surface were trafficked to an endosome. Following clathrin pit formation, internalized cargoes converge on the early endosome prior to being sorted for recycling or degradation. The early endosome is enriched with the Rab GTPase, Rab5a. Sorting to the early endosome has been implicated in HCV entry because a dominant negative Rab5 inhibits HCVpp entry [29].

**Figure 9:**
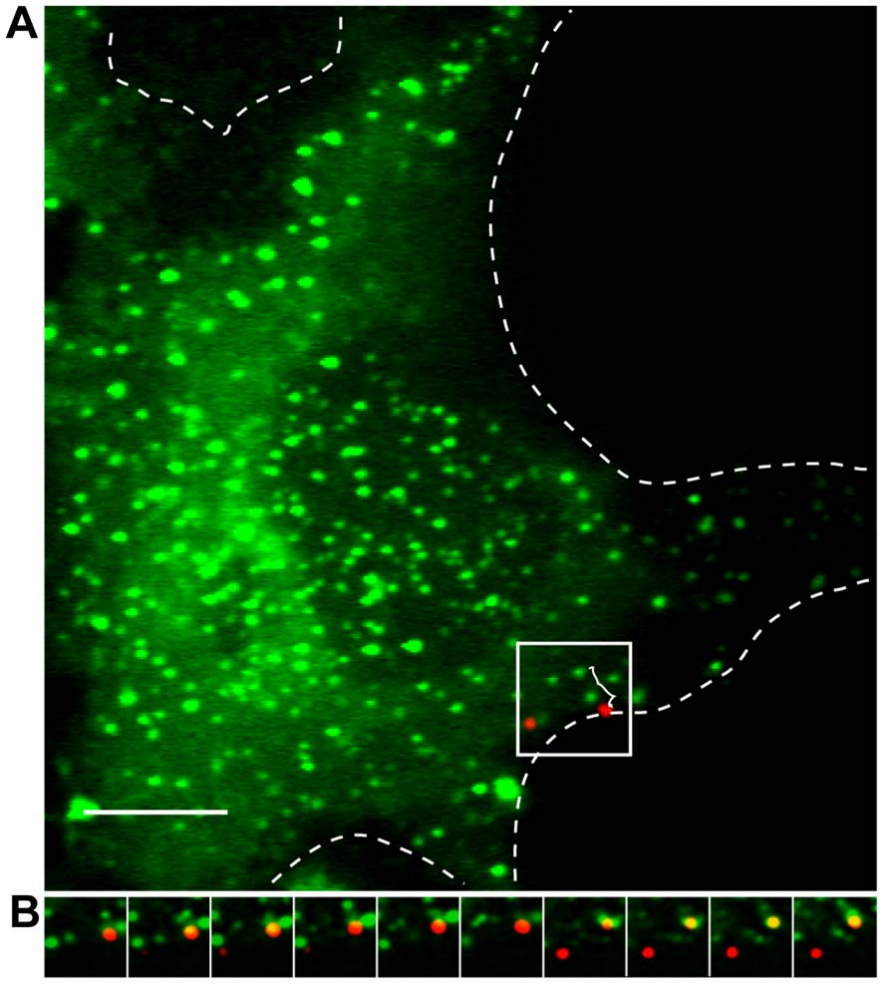
HCV is transported in Rab5a-containing early endosomes. A. Live cell imaging of Huh-7.5 cells transfected with GFP-Rab5a prior to imaging and infected with DiD-HCV for 1 hour on ice then shifted to 37°C upon imaging. Alternating Cy5 (DiD-HCV) and GFP (GFP-Rab5a) 100 ms exposures were taken every 2 seconds for several minutes. Edge of cell outlined. Trajectory traveled in white. B. Inset of A shown as montage of alternating Cy5 and GFP exposures. Scale bar = 5 µm.

We infected Huh-7.5 cells transiently expressing GFP-Rab5A on ice for one hour, shifted to 37°C and began imaging for 90 minutes. In Figure 9, a DiD-HCV particle transported on the cell surface before becoming associated with a GFP-Rab5a vesicle. This internalization occurred outside of cell-cell junctions, indicating that delivery of DiD-HCV to early endosomes does not require virion entry at a tight junction.

## Discussion

In this study, we combined an unbiased RNAi analysis that identified 16 cellular proteins involved in HCV endocytosis with the first description of single particle tracking of HCV infection. This approach enabled us to identify new cellular cofactors of HCV infection and to image the trafficking of HCV in relation to these cellular proteins during internalization. In doing so, we have confirmed the importance of clathrin-mediated endocytosis while identifying many molecular details of the entry process. Additionally, our data indicate that the current model of HCV entry, at least into Huh-7.5 cells, requires modifications and refinement.

We propose the following model for HCV entry into Huh-7.5 cells based on our data and other studies. HCV initially relies on actin-based transport by binding to filopodia and trafficking with retrograde actin flow to reach the cell surface. The viral particle diffuses randomly until the engagement of receptor molecules CD81 and SR-B1. This complex moves laterally until encountering the entry co-factors claudin-1, -6 or -9, and possibly occludin, typically in a region not associated with tight junctions. Once the virus and receptor complex are associated with clathrin coated pits, the ubiquitin E3 ligase c-Cbl, which may ubiquitylate one of the co-receptors, is associated with subsequent virion internalization and sorting into endosomes. The clathrin coat is linked to the actin cytoskeleton by specialized adapter molecules epsins 1 or 3 and endocytosis is linked with clathrin coated pit curvature, fission, and actin nucleation. Following internalization, the virion migrates along actin stress fibers and the ubiquitylated receptor-HCV complex is sorted by Hgs into appropriate endosomal compartments. Intracellular trafficking of early endosomes away from the plasma membrane relies on actin-based transport along stress fibers. The virus remains in the endosome until an internal pH is achieved that stimulates fusion occurs between the viral envelope and the endosomal membrane, which is presumably facilitated by the vacuolar ATPase, ATP6v0a1.

Our findings propose multiple roles for actin in HCV entry, including early interactions with filopodial actin, actin nucleation during internalization, and migration with actin stress fibers following HCV internalization. Filopodial actin-based transport of virions to the cell surface, where the entry receptors are clustered, has been previously described for other viruses [49],[50]. The cortical actin cytoskeleton serves to provide structural plasticity to a cell and also serves as a barrier for entry of a virus into a cell. To get around this, many viruses trigger actin stress fiber breakdown for efficient viral entry [57],[58]; however, we found HCV to be associated with or transport along intact stress fibers. Actin stress fibers have been suggested to increase during HCV infection [27], but we did not see an obvious increase in stress fibers associated with DiD-HCV infection (data not shown).

A topic that we are actively pursuing is the precise role of c-Cbl in HCV entry. c-Cbl is an ubiquitin E3 ligase that was been implicated in the internalization of receptor molecules and receptor tyrosine kinase signaling. Additionally, c-Cbl is implicated in the receptor-mediated endocytosis of the bacterial pathogens *Rickettsia conorri* and *Lysteria monocytogenes* [59],[60]. The ubiquitin ligase, CBLL1, is implicated in the receptor-mediated entry of another member of the *Flaviviridae*, West Nile virus [61]. Our data support a similar role in HCV infection given that c-Cbl is required for HCV entry and that it localizes with HCV particles with similar kinetics as the clathrin light chain. Of particular interest is the identity of the ubiquitylated receptor and investigating whether c-Cbl-dependent signal transduction pathways are required for HCV entry.

The data presented here suggest that HCV entry does not require tight junctions. This is supported by another study, which reports that disruption of tight junctions by calcium depletion enhances HCVpp infection of polarized Caco-2 and HepG2 cells [62],[63]. Nevertheless, we think it is likely that HCV infection *in vivo* is likely to occur at the tight junction, given that claudin-1 and occludin localization is thought to be limited to the tight junction in primary polarized hepatocytes [64]. One wonders why HCV has evolved such a complex series of receptor interactions, including two tight junction proteins, when tight junctions are dispensable for entry in cell culture. We would suggest that the driving evolutionary force for HCV to use tight junction receptor proteins is primarily to promote cell-cell spread of the virus. HCV infection in cell culture appears to rely on cell to cell spread, in addition to extra-cellular infection [65]. Cell-cell spread is independent of the virus receptor CD81, but is dependent on claudin-1 [65],[66]. Thus, HCV may tolerate a complicated, inefficient initial entry process that is compensated by efficient cell-cell spread of the virus. The development of robust polarized cell culture models of HCV infection that can be used with live cell imaging will shed light on these questions.

We believe that these data may have an impact in HCV therapeutic development. HCV entry is an attractive target for small molecule inhibitors since it precedes the assembly of infectious virus and the maintenance of chronic infection is likely to required continual rounds of re-infection of hepatocytes. The 16 host cofactors of HCV infection identified in this study include a number of enzymes that may be successfully targeted by pharmacological inhibitors. The development of live cell imaging of HCV infection greatly expands our ability to study dynamic HCV-cellular interactions. Additionally, it will be an important approach to characterizing the mechanism of action of HCV entry inhibition by small molecules and neutralizing antibodies.

## Materials and Methods

### Cells and virus

Huh-7.5 cells [67] were grown in Dulbecco's modified high glucose media (DMEM; Invitrogen) supplemented with 10% fetal bovine serum (FBS; Invitrogen), nonessential amino acids (NEAA, 0.1 mM; Gibco), 1% penicillin-streptomycin (Gibco), and maintained in 5% CO_2_ at 37°C. For transfection of viral RNA, Huh-7.5 cells were electroporated with HCV genotype 2a infectious clone pJFHxJ6-CNS2C3 RNA as described in [33],[68]. Viral supernatants were collected daily from up to 5 passages of the electroporated cells, filtered through a 0.22 micron cellulose nitrate filter, and kept at 4°C protected from light. Viral titers were determined by limiting dilution and immunohistochemical staining using an antibody directed to NS5A (9E10) as described [7],[69].

### Highly infectious stock preparation

Viral supernatants were concentrated by polyethylene glycol 8000 (PEG) precipitation [7]. Briefly, filtered viral supernatants were incubated overnight with PEG (final, 8% (w/v)) followed by centrifugation (8000×g, 20 min) and resuspended in 10 mL of spent supernatant. Virus was centrifuged (8000×g, 15 min) and resuspended in a final volume of 1 mL of cell culture DMEM. Virus was labeled by adding 5 µL (5 µM final concentration) of lipophilic dye, DiD (Invitrogen, excitation 644 nm/emission 665 nm) or DiI (Invitrogen, excitation 549 nm/emission 565 nm) to the 1 mL of concentrated virus. Virus and dye were incubated for 1 hour with shaking at room temperature while protected from light. Labeled virus was loaded onto a 20–80% (w/v) continual sucrose gradient (in phospho-buffered saline (PBS)) and centrifuged for 16 hours at 4°C and 34K rpm. 1 mL fractions were isolated after puncture of the bottom of the tube. Each fraction was titered to determine an infectious dose 50 (ID_50_)/mL by immunohistochemical staining as described above. Viral RNA was isolated by Trizol-LS (Invitrogen) extraction from 100 µL of each fraction. The copies of viral RNA present in each fraction were determined by quantitative real time reverse transcriptase (RT) PCR analysis as described previously [33]. RT-PCR was performed using an Applied Biosystems 7300 Real Time PCR machine under the following conditions: 30 min at 70°C, 6 min at 95°C, then cycled 50 times at 95°C for 15 sec, 55°C for 30 sec, 72°C for 15 sec. The absolute RNA copy number was determined for each isolated fraction by comparison to viral RNA concentration standards. The specific infectivity per RNA copy of each fraction was determined by dividing the RNA quantity by the ID50/mL. DiD-HCV was isolated from the sucrose for subsequent imaging studies by ultracentrifugation at 36K rpm for 2 hours, and then resuspended in 1 mL of DMEM supplemented with 10% FBS, 0.1 mM NEAA, and 1% penicillin-streptomycin. Gradient fractions that contain DiD-HCV with specific infectivities of ∼10, such as fraction 5 in Figure 1, were used for imaging studies.

### Immunodepletion of DiD-HCV

100 µL fractions of DiD-HCV were incubated with 10 µg of E2 (CBH5) or isotype control (RO4) antibodies (generous gifts from Steven Foung) already bound to IgG beads (Sigma) for 1 hour with shaking at 4°C. Beads were spun down by centrifugation at 12000×g for 5 minutes at 4°C. Unbound particles in the supernatant were removed using a 28G needle. 10 µL of supernatant or input DiD-HCV fraction were spotted onto poly-lysine coverslips and immuno-stained as described below.

### Drug inhibitor studies

Cytochalasin D (Sigma) was kept at a stock concentration of 0.5 mg/mL in DMSO at -20°C and diluted in 5% DMEM for inhibitor studies. Cells were seeded in a 96 well plate and treated for approximately 1 hour with indicated concentrations of cytochalasin D, then infected with pseudotyped HCV viral particles in drugged media. Viral inoculum was replaced with fresh 5% DMEM approximately 4 hours later and then cells were incubated for 48 hours. After 48 hours, the cell lysates were harvested and tested via luciferase assay (Promega).

For the DiD-core uncoating assay in the presence of NH_4_Cl, Huh-7.5 cells grown on 13 mm round coverslips were incubated with 100 mM NH_4_Cl for 2 hours at 37°C. Cells were incubated on ice for 10 minutes before the addition of DiD-HCV (MOI = 1) for 1 hour on ice. NH_4_Cl was present throughout the duration of the experiment.

### Cell viability assay

Viability was assessed using the CellTiter-Glo luminometer viability assay as directed by the manufacturer (Promega).

### Plasmid constructs

CD81 was PCR amplified from Huh7.5 cDNA with primers (5′-GGATCCATGGAGTGGAGGGCTG-3′, 5′-GGATCCGTACACGGAGCTGTT CC-3′) to allow insertion of *BamHI* restriction sites (underlined sequence) flanking the gene. Rab5A was cloned as N-terminal GFP fusion in pmGFP-C1 with a 5′ *HindIII* site engineered into the forward primer and a 3′ *KpnI* site engineered into the reverse primer: (F: 5′- GCAAGCTTCAACCATGGCTAGTCGAGGCGCAA-3′, R: 5′- ACGGTACCTTAGTTACTACAACACTGATTCCT-3′). All plasmids were confirmed by sequence analysis using an Applied Biosciences 3730XL sequencer. The CD81 PCR product was initially cloned into a pCR 2.1-TOPO plasmid (Invitrogen) via the manufacturer's instructions and then subcloned into pTRIP-GFP (an HIV-based transfer vector) [70],[71] to result in the CD81-GFP plasmid. The Rab5a PCR product was cloned directly into pmGFP-C1, resulting in the plasmid GFP-Rab5a. The sequence, gene orientation and frame were confirmed via sequencing. The GFP-actin construct was a generous gift from Jerold Turner (University of Chicago).

### Lentivirus production

To produce CD81-GFP lentiviral stocks, 293T cells were transfected with the following plasmids: a plasmid containing the HIV *gag* and *pol* genes, and a plasmid containing the vesicular stomatitis virus glycoprotein (VSV-G), and CD81-GFP plasmids. Plasmids were transfected into 293T cells using Lipofectamine 2000 per the manufacturer's instructions. Supernatants containing the pseudotyped virus was harvested after 48 hours at 37°C, passed through a 0.45µM filter, aliquoted, and stored at −80°C.

### RNA interference assay

The primary screen included siRNAs that were designed to target 140 genes (4 siRNAs per gene) (Dharmacon, Inc.). Genes important for infectious HCV production were subsequently silenced with four individual siRNAs targeting the gene to eliminate the possibility of the phenotype being associated with off target effects. RNAi assays were performed as previously described [35],[72]. Briefly, 1×10^6^ Huh-7.5 cells in 0.05 ml of PBS pH 7.4 were electroporated with 125 picomoles of siRNA for 5 pulses of 770 volts for 99 microseconds with one- second intervals on a BTX 830 electroporator with 96-well attachment. Cells were plated and infected at 72 hours after electroporation with a multiplicity of 0.5 infectious HCV particles per cell for 6 hours, rinsed with media, then maintained for 2 days at 37°C. After infection, the supernatants were collected and analyzed for extra-cellular infectious virus via limiting titer dilution as described previously [69].

For the uncoating assay in siRNA expressing cells, 1×10^6^ cells were transfected with 125 pmol of either siIRR and siCD81 constructs using Lipofectamine RNAi max reagent (Invitrogen). siRNAs were allowed to express for 48 hours before the cells were seeded onto 13 mm round coverslips. Cells were infected for the uncoating assay and fixed and stained using a core antibody as described below. The silenced cells were also tested in the HCV pseudoparticle entry assay described below.

### HCV pseudoparticle entry assay

HCVpp were generated as described previously [33]. Huh-7.5 cells were infected 72 h after siRNA electroporation with HCVpp and 8 µg/ml polybrene (Sigma) for 4 h. Cell lysates were collected at 48 h after HCVpp infection, and firefly luciferase (Promega) activity was measured using a 96-well luminometer (Centro LB 960, Berthold Technologies).

### Live cell imaging

Huh-7.5 cells were transfected with GFP-Rab5a or GFP-actin constructs using Lipofectamine 2000 (Invitrogen) as per manufacturer's instructions. After 24 hours of expression transfected cells were seeded onto poly-lysine coated 35 mm fluorodishes (World Precision Instruments) at a confluency of 60–70% and allowed to adhere for 24 hours prior to imaging. Alternatively, Huh-7.5 cells were infected with CD81-GFP lentivirus in the presence of 1 µg/mL polybrene and allowed to establish expression for 48 hours followed by seeding onto fluorodishes as above. Cells were washed with PBS and 100 µL of virus diluted in 400 µL of DMEM was added to each dish on ice and protected from light. Experiments were performed at MOIs of 0.2 to 1.0, dependent on the calculated specific infectivity of the purified DiD-HCV stock, which yielded on average 4 DiD-HCV particles per cell. After 1 hour, the media was replaced with fresh cold 10% FBS DMEM containing 25 mM HEPES buffered saline (Gibco). The dish was placed on a heated stage at 37°C on an Olympus DSU Spinning Disc confocal microscope.

Cells were visualized using a 100×1.45 N.A oil-immersion objective. DiD-HCV or DiI-HCV particles were visualized using a Cy5 or Cy3 filter set, respectively. GFP-Rab5a, GFP-actin, or CD81-GFP proteins were visualized using an EGFP filter set coupled to a Hamamatsu back-thinned EM-CCD high speed/sensitivity camera. Images were acquired by taking sequential Cy5/EGFP exposures at 100 ms every 5 seconds for 50-100 frames up to 2 hours post temperature shift with the camera intensification set at 255 using the Slidebook software. Images were processed using the Image J software (National Institutes of Health). Trajectories and corresponding velocities were determined using the Image J plug-in, SpotTracker [73]. Mean square displacement was determined using the following equation:

MSD*(τ)* = 〈(*x(t)-(x(t+τ)*)*^2^+*(*y(t)-(y(t+τ)*)^2^〉

Where *x* and *y* are the coordinates of the particle, *τ* is the lag time, and the brackets represent the time average [74].

### Immunofluorescence microscopy

Huh-7.5 cells were seeded onto 13 mm round coverslips at a confluency of 60–70%. Cells were washed once with ice cold PBS and infected with highly infectious DiD-HCV at a MOI of 0.5 on ice for 1 hour and protected from light. Unbound virus was washed off with cold PBS, DMEM+5%FBS media was added, and cells were transferred to the 37°C incubator. Coverslips were fixed in paraformaldehyde at 0 min, 15 min, 30 min, 1 hour, 1.5 hour, and 2 hours post temperature shift and kept at 4°C until processed. Coverslips were permeabilized with 0.1% Triton X-100 and blocked in 0.1% Triton X-100-50% goat serum in PBS. Coverslips were incubated overnight with primary antibodies as follows: 1∶100 anti-core (Virostat), 1∶200 anti-c-Cbl (Cell Signaling Antibodies), 1∶200 anti-clathrin light chain (Santa Cruz), 1∶500 anti ZO-1 (Zymed), and 1∶100 anti-claudin-1 (Zymed) in blocking buffer. Following primary antibody incubation, coverslips were washed two times with PBS and incubated with Alexa Fluor 488 conjugated secondary antibody (1∶1000) for 1 hour before mounting on either Mowiol (Sigma) or Prolong Gold (Invitrogen) reagent. Actin was stained using Alexa Fluor 488 phalloidin (Invitrogen) at 1∶40 in PBS following paraformaldehyde fixation.

For immuno-staining of DiD-HCV particles for viral proteins, particles were aliquoted onto poly-lysine treated coverslips and fixed in 4% paraformaldehyde and permeabilized in 50% goat serum plus 0.1% TX-100. DiD-HCV particles were incubated with 1∶100 anti-core (Virostat) or 1∶100 anti-E2 (CBH5) for 1 hour. Following primary antibody incubation, coverslips were washed and incubated with fluorescent conjugated secondary antibody as above.

Coverslips were imaged for DID labeled virus by using the Cy5 filter set and Alexafluor 488 was visualized using the GFP filter set. Immunofluorescence images were taken using an Olympus DSU Spinning Disc Confocal microscope with a 100X NA 1.45 objective where the camera intensification was set to 255. DiD-HCV images were acquired at an exposure time of 1 second using the Cy5 filter set and DAPI stained nuclei were visualized at an exposure time of 20 ms using the 350 filter set. Alexa fluor 488 conjugated secondary antibodies were detected using the EGFP filter set and the following exposure times: clathrin light chain 1 second, C-Cbl, 1 second, CLDN-1 and ZO-1, 500 ms. Post-acquisition, images were processed using Image J (NIH). Specifically, each red, green, and blue stack was separated into individual channels. Each channel was processed by using the subtract background tool followed by the smooth option. Channels were merged into a single image and the contrast was adjusted. For colocalization analysis two Image J plugins were used. The first, RGB profiler (Christophe Laummonerie), provided intensity profiles of each channel corresponding to a line drawn on an image. Colocalization was confirmed when peaks above background overlapped. The Image J plugin, colocalization, was also used to confirm overlapping pixels in the separate channels.

## Supporting Information

Figure S1 Cell viability following treatment with indicated siRNAs. Cell viability was determined five days after electroporation of siRNAs and quantified by a luminescence-based cell viability assay (Promega) that measures intracellular ATP levels. Values are measured relative to IRR siRNA-treated cells and standard error of the mean is shown.
(0.03 MB PDF)

Table S1 Genes and siRNAs tested in the RNA interference screen.
(0.10 MB PDF)

Table S2 Quantitative real time RT-PCR assays.
(0.05 MB PDF)

Video S1 Transport of DiD-HCV (red) along filopodia labeled with GFP-actin (green). Montage of video is shown in Figure 4A. Playback time is 10× real speed. Scale bar = 10µm.
(6.31 MB MOV)

Video S2 Diffuse movement of DiD-HCV on the cell surface. Video of the montage shown in Figure 4B. Playback time is 10× real speed. Scale bar = 10µm.
(4.37 MB MOV)

Video S3 Actin nucleation adjacent to a DiD-HCV particle. DiD-HCV leaves the focal plane, indicating an endocytic event. Video of the montage shown in Figure 4C. Playback time is 10× real speed. Scale bar = 10µm.
(0.70 MB MOV)

Video S4 DiD-HCV particle is transported by the nucleation of an asscociated actin cluster. Video of the monatge shown in Figure 4D. Playback time is 10× real speed. Dimensions of video are 8.48×10.08 µm.
(0.06 MB MOV)

Video S5 Actin depolymerization disrupts directional movement of DiD-HCV particles. Video of the montage shown in Figure 5C. Shown is a representative image of GFP-actin expressing cells treated with 2µM Cytochalasin D during live cell imaging acquisition of DiD-HCV entry. Cytochalasin D was added at 850 seconds. Image plane was readjusted after addition of Cytochalasin D to the imaging chamber and imaging took place for another 365 seconds. Playback time is 10× real speed. Scale bar = 10µm.
(0.17 MB MOV)

Video S6 Dil-HCV associates with CD81-GFP during entry into cells. Video of the montage shown in Figure 7B. DiI-HCV is associated with CD81-GFP on the cell surface, then enters into the cell. Internalization occurs away from the cell-cell junction. Playback time is 10× real speed. Scale bar = 10µm.
(4.72 MB MOV)
