# Supplementary figures and images for: RNA Interference and Single Particle Tracking Analysis of Hepatitis C Virus Endocytosis
Source: PLoS Pathog. 2009 Dec 24;5(12):e1000702. doi: 10.1371/journal.ppat.1000702 (PMC2790617; doi:10.1371/journal.ppat.1000702)

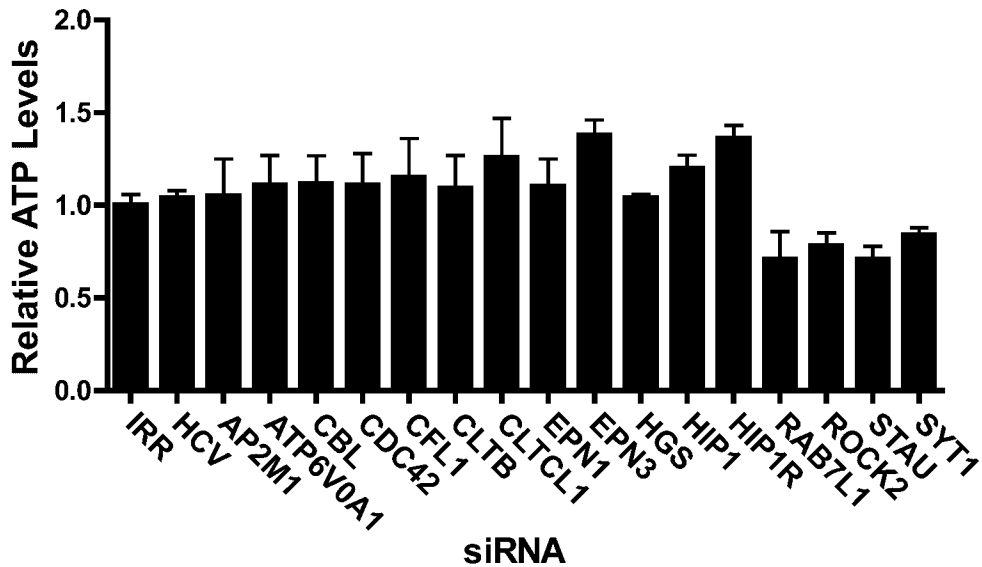

Supplement: Figure S1 — Cell viability following treatment with indicated siRNAs. Cell viability was determined five days after electroporation of siRNAs and quantified by a luminescence-based cell viability assay (Promega) that measures intracellular ATP levels. Values are measured relative to IRR siRNA-treated cells and standard error of the mean is shown. (0.03 MB PDF) [file ppat.1000702.s001.pdf]
