# Supplementary material for: RNA Interference and Single Particle Tracking Analysis of Hepatitis C Virus Endocytosis
Source: PLoS Pathog. 2009 Dec 24;5(12):e1000702. doi: 10.1371/journal.ppat.1000702 (PMC2790617; doi:10.1371/journal.ppat.1000702)
Supplement: Table S1 — Genes and siRNAs tested in the RNA interference screen. (0.10 MB PDF) [file ppat.1000702.s002.pdf]

SI Table 1. Genes and siRNAs tested in RNA interference screen.

Gene	GenBank Accession No.	Pooled siRNAs siGENOME SMARTpool (Primary Screen)	Individual siRNAs Catalog No.	Individual siRNAs Sequence
CLTCL1	NM_001835	GCAAAGUGAUCCAGUGUUU	D-011611-04 [a]	GAUUGGAGCUAAACAUGGU
		CCGAGUGGCUUGUCAAUUU	D-011611-17*	CGCCAAAGUUGCAGCGUCU
		CAGCCGACCUGCCUAAUGA		
		GAUUGGAGCUAAACAUGGU		
CLTB	NM_001834	GCGCCAGAGUGAACAAGUA	D-004003-03	GGAACCAGCGCCAGAGUGA
		GAAGGUGGCCCAGCUAUGU	D-004003-04	GAGCGAGAUUGCAGGCAUA
		GGAACCAGCGCCAGAGUGA		
		GAGCGAGAUUGCAGGCAUA		
HIP1R	NM_003959	CAGCUCAACUCGUGAACUA	D-027079-03	CCUCUUCGAUCAGACGUUU
		CUGUGGAGAUGUUUGAUUA	D-027079-04	CCGACAUGCUGUACUUCAA
		CCUCUUCGAUCAGACGUUU		
		CCGACAUGCUGUACUUCAA		
HIP1	NM_005338	GGAAGUGGCUGUAAAGGAA	D-005001-01	GGAAGUGGCUGUAAAGGAA
		GAACCAAGAUGGAGUACCA	D-005001-04	GAACCAAGAUGGAGUACCA
		GCAAAUCACAGAUCGAAGA		
		CUAAUGGUGUGUUCUCAUG		
EPN1	NM_013333	GGAAGACGCCGGAGUCAUU	D-004724-01	GGAAGACGCCGGAGUCAUU
		GAACUGGCGUCACGUUUAC	D-004724-02	GAACUGGCGUCACGUUUAC
		GGACCUUGCUGACGUCUUC	D-004724-03	GGACCUUGCUGACGUCUUC
		GAACGUGCGUGAGAAAGCU	D-004724-04	GAACGUGCGUGAGAAAGCU
EPN3	NM_017957	GCGAGAACCUCUACACCAU	D-021006-04	UGCCAAACCUCCAGAAUCC
		UAACAUUGCUGGACUACCU	132202 [b]	GCAAAAUGGCACGAAGGAG
		UCGCUGACCUGACCUUCAA	132200 [b]	CGUGUACAAGGCUCUAACA
		UGCCAAACCUCCAGAAUCC		
CFL1	NM_005507	UGACAGGGAUCAAGCAUGA	D-012707-01	UGACAGGGAUCAAGCAUGA
		GCGGUGCUCUUCUGCCUGA	D-012707-03	GUCAAGAUGCUGCCAGAUA
		GUCAAGAUGCUGCCAGAUA	D-012707-04	GCUAUGCCCUCUAUGAUGC
		GCUAUGCCCUCUAUGAUGC		
CDC42	NM_001791	GGAGAACCAUAUACUCUUG	D-005057-01	GGAGAACCAUAUACUCUUG
		GAUUACGACCGCUGAGUUA	D-005057-02	GAUUACGACCGCUGAGUUA
		GAUGACCCCUCUACUAUUG	D-005057-03	GAUGACCCCUCUACUAUUG
		CGGAAUAUGUACCGACUGU	D-005057-04	CGGAAUAUGUACCGACUGU
ROCK2	NM_004850	GAGGAAAGCUGAUCAUGAA	D-004610-02	GUAGAAACCUUCCCAAUUC
		GUAGAAACCUUCCCAAUUC	D-004610-03	GCAACUGGCUCGUUCAAUU
		GCAACUGGCUCGUUCAAUU	D-004610-05	GCAAAUCUGUUAAUACUCG
		GCAAAUCUGUUAAUACUCG	D-004610-18*	GCAGCAAUGGUAAGCGUAA
AP2M1	NM_004068	UAUAUGAGCUGCUGGAUGA	D-008170-01	UAUAUGAGCUGCUGGAUGA
		GAAGAGCAGUCACAGAUCA	D-008170-02	GAAGAGCAGUCACAGAUCA
		CGUGAUGGCUGCCUACUUU	D-008170-04	GGAGGCUUAUUCAUCUAUA
		GGAGGCUUAUUCAUCUAUA		
SYT1	NM_005639	GCAAUUUACUUUCAAGGUA	D-020044-01	GCAAUUUACUUUCAAGGUA
		GUAAGAGGCUGAAGAAGAA	D-020044-02	GUAAGAGGCUGAAGAAGAA
		GAUCGUUUCUCUAAGCAUG	D-020044-03	GAUCGUUUCUCUAAGCAUG
		CGACUGUUCUGCCAAGCAA		
CBL	NM_005188	GGAGACACAUUUCGGAUUA	s2477 [b]	GAUUGAUAGCUGUACGUAU

		GAUCUGACCUGCAAUGAUU<br>GACAAUCCCUCACAAUAAA<br>CCAGAAAGCUUUGGUCAUU	121529 [b]	CCUUAUAUCUUAGACCUGCC
HGS	NM_004712	GCACGUCUUUCCAGAAUUC<br>AGAGAGCGAUGCCAUGUUU<br>GAUAUUCUGUGGAAAGUGU<br>GUAAACGUCCGUAACAAGA	137616 [b]<br>103410 [b]	CGACAAGAACCCACACGUC<br>GGACCUGCUGAAGAGACAA
ATP6V0A1	NM_005177	GAAGAUGUCUGUUAUCCUU<br>GGAAGAGGCACUCCUUUAA<br>CCAAUAAACUGACGUUCUU	D-017618-01<br>D-017618-04	GAAGAUGUCUGUUAUCCUU<br>GAACUUACCGAGAGAUAAA
STAU	NM_004602	AAACGGAACUUGCCUGUGA<br>GAACGAAUUUGUAUCUCUU<br>GCAAUUUAAUGGCAAAGGA<br>CACGGUAACUGCCAUGAUA	D-011894-02<br>D-011894-03	GAACGAAUUUGUAUCUCUU<br>GCAAUUUAAUGGCAAAGGA
RAB7L1	NM_003929	CAGAUUGACCGGUUCAGUA<br>GAGAACGGUUUCACAGGUU<br>GGGACUACAUCAAUCUACA<br>CAACAAGUGUGAUCUGUCC	D-010556-01<br>D-010556-03<br>D-010556-05<br>D-010556-06	CAGAUUGACCGGUUCAGUA<br>GAGAACGGUUUCACAGGUU<br>GGGACUACAUCAAUCUACA<br>CAACAAGUGUGAUCUGUCC
ACACA	NM_198834	M-004551-02 [a]		
ACTR2	NM_005722	M-012076-00		
ACTR3	NM_005721	M-012077-01		
ADAM10	NM_001110	M-004503-01		
AMPH	NM_001635	M-011569-00		
AP1B1	NM_001127	M-011200-00		
AP1M1	NM_032493	M-013196-00		
AP1M2	NM_005498	M-012056-00		
AP2A1	NM_014203	M-012492-00		
AP2A2	NM_012305	M-012812-00		
AP2B1	NM_001282	M-003627-01		
ARF1	NM_001658	M-011580-00		
ARF3	NM_001659	M-011581-00		
ARF5	NM_001662	M-011584-00		
ARF6	NM_001663	M-004008-00		
ARFIP2	NM_012402	M-012820-00		
ARPC1B	NM_005720	M-012082-01		
ARPC2	NM_005731	M-012081-00		
ARPC3	NM_005719	M-005284-00		
ARPC4	NM_005718	M-008571-00		
ARPC5	NM_005717	M-012080-00		
ARRB1	NM_004041	M-011971-00		
ARRB2	NM_004313	M-007292-00		
ATG5	NM_004849	M-004374-03		
ATG12	NM_004707	M-010212-02		
ATM	NM_138293	M-003201-02		
BIN1	NM_004305	M-008246-00		
CAMK1	NM_003656	M-004940-00		
CAV1	NM_001753	M-003467-01		
CAV2	NM_001233	M-010958-00		
CAV3	NM_001234	M-011229-00		

CBLB	NM_170662	M-003004-01
CIB1	NM_006384	M-012261-00
CIB2	NM_006383	M-012230-00
CIB3	NM_054113	M-012901-00
CLTA	NM_001833	M-004002-00
CLTC	NM_004859	M-004001-00
COPA	NM_004371	M-011835-00
COPB2	NM_004766	M-019847-01
CTBP1	NM_001328	M-008609-01
DAB2	NM_001343	M-008522-00
DDEF2	NM_003887	M-011544-00
DIAPH1	NM_005219	M-010347-01
DNM1	NM_004408	M-003940-00
DNM2	NM_004945	M-004007-01
DNM3	NM_015569	M-013931-00
EEA1	NM_003566	M-004012-01
EFS	NM_005864	M-012094-00
ELKS	NM_015064	M-010942-00
ENTH	NM_014666	M-021406-00
EPN2	NM_148921	M-004725-00
EPS15	NM_001981	M-004005-00
EPS15L1	NM_021235	M-004006-00
FASN	NM_004104	M-003954-04
FYN	NM_002037	M-003140-03
GAF1	NM_015470	M-004298-00
GIT1	NM_014030	M-004298-00
GNB2L1	NM_006098	M-006876-00
GORASP1	NM_031899	M-013510-00
GRB2	NM_002086	M-019220-00
IHPK3	NM_054111	M-006739-00
ITSN1	NM_003024	M-008365-00
ITSN2	NM_006277	M-009841-00
LIMK1	NM_002314	M-007730-01
MAP4K2	NM_004579	M-003587-01
MAPK8IP1	NM_005456	M-003595-00
MAPK8IP2	NM_012324	M-012462-00
MAPK8IP3	NM_015133	M-003596-01
NEDD4	NM_006154	M-007178-01
NEDD4L	NM_015277	M-007187-01
NSF	NM_006178	M-009401-00
PACSIN1	NM_020804	M-007735-00
PACSIN3	NM_016223	M-015343-00
PAK1	NM_002576	M-003521-03
PICALM	NM_007166	M-004004-02
PIK3C2G	NM_004570	M-006773-00
PIK3CG	NM_002649	M-005274-02
PIK4CA	NM_002650	M-006776-03
PIK4CB	NM_002651	M-006777-02
PIP5K1A	NM_003557	M-004780-02
PITPNM1	NM_004910	M-019888-00

PRKCM	NM_002742	M-005028-00
PSCD3	NM_004227	M-019268-00
RAB11A	NM_004663	M-004726-02
RAB11B	NM_004218	M-004727-01
RAB3A	NM_002866	M-009668-01
RAB3B	NM_002867	M-008825-00
RAB3C	NM_138453	M-008520-00
RAB3D	NM_004283	M-010822-01
RAB4A	NM_004578	M-008539-01
RAB4B	NM_016154	M-008780-01
RAB5A	NM_004162	M-004009-00
RAB5B	NM_002868	M-004010-01
RAB5C	NM_004583	M-004011-01
RAB6A	NM_002869	M-008975-01
RAB6B	NM_016577	M-008548-00
RAB7B	NM_177403	M-018225-00
RAB8A	NM_005370	M-003905-00
RAB8B	NM_016530	M-008744-00
RAC1	NM_018890	M-003560-02
RHOA	NM_001664	M-003860-00
ROCK1	NM_005406	M-003536-01
SARA1	NM_020150	M-016756-00
SH3GLB1	NM_016009	M-017086-00
SH3GLB2	NM_020145	M-015810-00
SNAP91	NM_014841	M-032296-00
SREBF1	NM_004176	M-006891-00
SYNJ2	NM_003898	M-012624-00
SYT2	NM_177402	M-018809-00
TNIK	XM_039796	M-004542-02
VAMP1	NM_014231	M-012497-00
VAMP2	NM_014232	M-012498-00
VAPA	NM_003574	M-021382-00
VAPB	NM_004738	M-017795-00
VAV2	NM_003371	M-005199-00
VIL2	NM_003379	M-017370-01
WAS	NM_000377	M-028294-01
WASF1	NM_003931	M-011557-00
WASF2	NM_006990	M-012141-00
WASF3	NM_006646	M-012301-01

[a] Dharmacon product number

[b] Applied Biosystems siRNA ID #

* siRNA not part of original SMARTpool for primary screen
