# Supplementary material for: RNA Interference and Single Particle Tracking Analysis of Hepatitis C Virus Endocytosis
Source: PLoS Pathog. 2009 Dec 24;5(12):e1000702. doi: 10.1371/journal.ppat.1000702 (PMC2790617; doi:10.1371/journal.ppat.1000702)
Supplement: Table S2 — Quantitative real time RT-PCR assays. (0.05 MB PDF) [file ppat.1000702.s003.pdf]

SI Table 2. Quantitative real time RT-PCR assays

Target	PCR Primer-Probe Sets[a]
CLTCL1	Hs00233589_m1
CLTB	Hs01126328_m1
HIP1R	Hs00391321_m1
HIP1	Hs00193477_m1
EPN1	Hs00203391_m1
EPN3	Hs00978957_m1
CFL1	Hs00830568_g1
CDC42	Hs00741586_mH
ROCK2	Hs00178154_m1
AP2M1	Hs01037584_m1
SYT1	Hs00194572_m1
HGS	Hs00610371_m1
ATP6V0A1	Hs00193110_m1
RAB7L1	Hs00187510_m1
GAPDH	4326317E
18S	4319413E

[a] Applied Biosystems product no.
